# Exploring the Relationship of SARS-CoV-2 and Uric Acid Levels With a Focus on Gout Patients: A Scoping Review

**DOI:** 10.7759/cureus.57138

**Published:** 2024-03-28

**Authors:** Hemangi Patel, Mahi Basra, Rohit Muralidhar, Michelle Demory Beckler, Marc M Kesselman

**Affiliations:** 1 Sports Medicine, Nova Southeastern University Dr. Kiran C. Patel College of Osteopathic Medicine, Fort Lauderdale, USA; 2 Osteopathic Medicine, Nova Southeastern University, Clearwater, USA; 3 Medicine, Nova Southeastern University Dr. Kiran C. Patel College of Osteopathic Medicine, Fort Lauderdale, USA; 4 Microbiology and Immunology, Nova Southeastern University Dr. Kiran C. Patel College of Allopathic Medicine, Fort Lauderdale, USA; 5 Rheumatology, Nova Southeastern University Dr. Kiran C. Patel College of Osteopathic Medicine, Davie, USA

**Keywords:** rheumatic disorder, gout disease, sars-cov-2 (severe acute respiratory syndrome coronavirus -2), gout, sars-cov-2

## Abstract

Rheumatic diseases are a group of conditions including arthritis and various other conditions that can lead to chronic inflammation within the musculoskeletal system, which can have negative effects on soft tissues, bones, muscles, joints, and connective tissue. One form of arthritis is gout, which is an inflammatory condition in which urate acid crystals build up in joints. Gout is associated with joint swelling, pain, redness, and joint mobility issues. Early diagnosis and treatment are essential to prevent joint degradation and other adverse complications. The condition has been shown to increase the incidence of diseases outside the musculoskeletal system, including the renal and cardiovascular systems. Comorbid conditions associated with gout include but are not limited to type 2 diabetes mellitus (T2DM), hypertension, hyperlipidemia, chronic kidney disease, cardiovascular disease, and heart failure. This systematic review aims to provide insight into the relationship between severe acute respiratory syndrome coronavirus 2 (SARS-CoV-2) infection, uric acid levels, and gout.

## Introduction and background

Rheumatic diseases are a group of conditions including arthritis and various other conditions that can lead to chronic inflammation within the musculoskeletal system, which can have negative effects on soft tissues, bones, muscles, joints, and connective tissue [1]. One form of arthritis is gout, which is a crystalline inflammatory joint disease that results in symptoms of episodic arthritis manifesting as gout flares. Gout is associated with joint swelling, pain, redness, and joint mobility issues. It most commonly affects older males and can be worsened by the consumption of alcohol, high fructose corn syrup, and protein, as well as the byproducts of chemotherapy treatment [2]. 

Gout occurs due to the inflammatory response driven by interleukin 1B in response to monosodium urate crystal deposition [2,3]. This process involves the accumulation of uric acid crystals in an individual’s joints, which initiates a primary inflammatory process. Phagocytic cells consume the uric acid crystals, which causes the release of enzymes from lysosomes and inflammatory cytokines including TNF, IL-1, IL-6 and IL-8 [2,3]. This results in endothelial cell dysfunction, which leads to vasodilation and increased vascular permeability. In addition, neutrophils are recruited as a result of cytokine release, especially as a result of the production of IL-8 [2]. This leads to chronic urate accumulation, which leads to tophi formation and results in joint destruction and deformity if untreated [2,3]. 

The condition has been shown to increase the incidence of diseases outside the musculoskeletal system, including the renal and cardiovascular systems. Comorbid conditions associated with gout include, but not limited to type 2 diabetes mellitus (T2DM), hypertension, hyperlipidemia, chronic kidney disease, cardiovascular disease, and heart failure [3,4]. The increase in uric acid levels and chronic inflammatory state tied to gout has been shown to play a role in viral infections, including severe acute respiratory syndrome coronavirus 2 (SARS-CoV-2) infection. While gout does not necessarily increase a patient’s risk of coronavirus disease (COVID)-19 infection, it has been shown to be associated with a higher risk of poor outcomes and higher mortality rates due to comorbid cardiovascular and renal conditions [1]. 

This can be explained, in part, by changes in uric acid levels. Specifically, the varying levels of uric acid in patients with gout when infected with SARS-CoV-2 can have implications on outcomes. Specifically, it has been shown that patients with gout who experience severe SARS-CoV-2 infection were found to have lower uric acid levels, while those patients with gout with mild SARS-CoV-2 infection had higher uric acid levels. Although gout is caused by increased levels of serum uric acid, some studies suggest that SARS-CoV-2 lowers uric acid levels. Other studies mention a U-shaped distribution. This change in uric acid is described in a U-shaped manner with the low being less than 278 µmol/L of serum uric acid and the high being greater than 423 µmol/L of serum uric acid [2]. As such, a measure of uric acid levels may serve as a prognostic indicator of the potential severity of SARS-CoV-2 infection among patients with gout. 

In addition, poor SARS-CoV-2 outcomes in patients with gout have been associated with increased serum levels of tumor necrosis factor a (TNF-a), interleukin-6 (IL-6) and interleukin-8 (IL-8) [5]. Furthermore, medications including colchicine used to treat gout have been shown to be associated with poor outcomes [6,7]. This systematic review aims to provide insight into the relationship between SARS-CoV-2 infection, uric acid levels, and gout.

Further, poor SARS-CoV-2 outcomes have been linked with increased serum levels of TNF-a, IL-6, and IL-8 [5]. Additionally, medications such as colchicine, which are used to treat gout, may affect patient outcomes in a positive manner [6,7]. This systematic review further aims to investigate the relationship and outcomes between SARS-CoV-2, gout and hyperuricemia.

## Review

Methods

Search Strategy and Selection Criteria

The Preferred Reporting Items for Systematic Reviews and Meta-Analyses (PRISMA) guidelines for performing systematic reviews by the Cochrane Collaboration were utilized. A literature search was conducted using three databases: EMBASE, PubMed, and Medline. Articles included must have been peer-reviewed, written in English, and performed in a human patient population between January 2013 and November 2023. The patient population must have been conducted in either a primary study, cohort study, cross-sectional study, or a clinical trial. Articles excluded from this review included review articles, not written in English, and animal studies. These criteria were selected to help fulfill the goals and objectives of this review. 

*Key Terms*

The key terms used to search for articles were gout, gouty arthritis, uric acid, inflammatory arthritis, coronavirus, COVID-19, COVID-19, and SARS-COV. Databases were searched using the Boolean operators “AND” and “OR” as follows: (“gout*” OR (“gouty arthritis*” OR “uric acid*” OR “inflammatory arthritis*”)) AND (“coronavirus*” OR “COVID-19*” OR “COVID*” OR “SARS-COV*“)). This search was conducted on November 2, 2023. 

Evaluation Process

**Figure 1:**
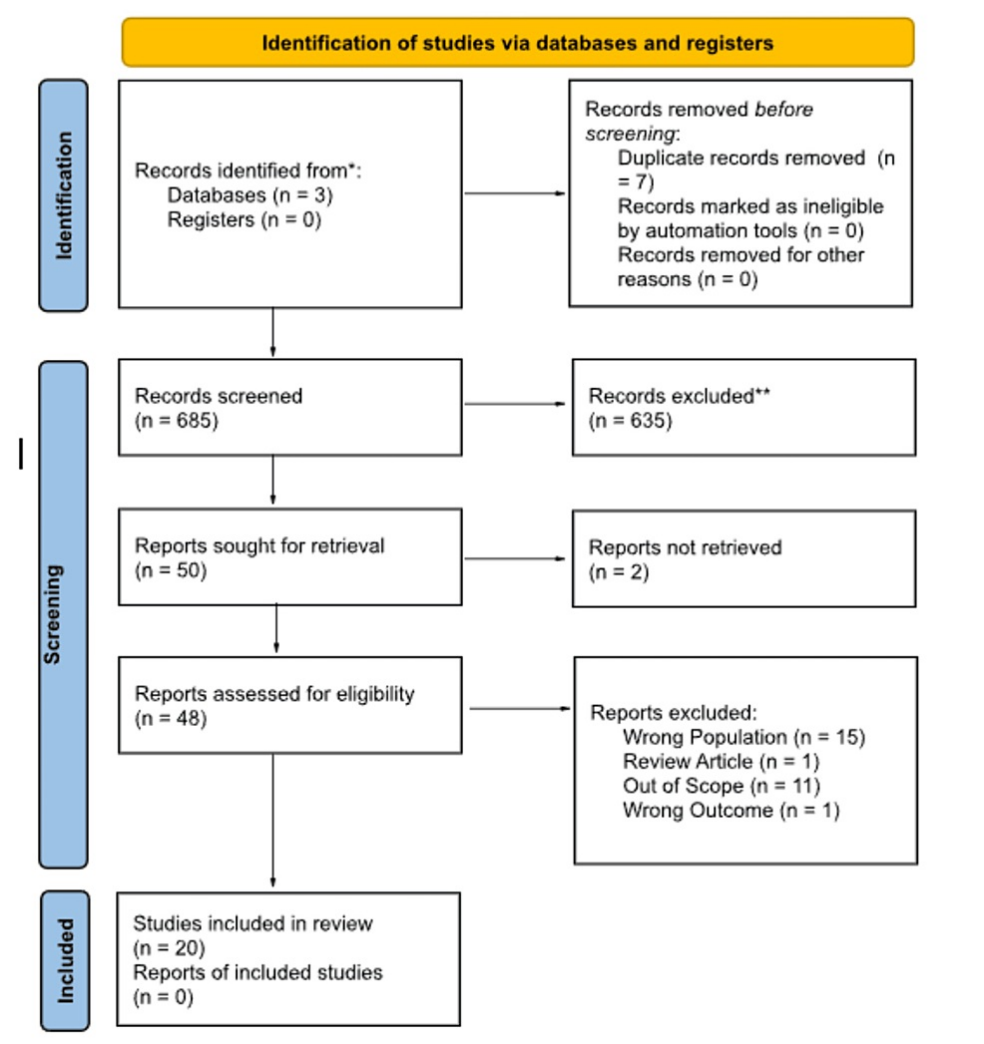
Flow diagram summarizing the literature screening process, based on the PRISMA 2020 template. PRISMA: Preferred Reporting Items for Systematic Reviews and Meta-Analyses.

The process of inclusion for the articles is depicted in the PRISMA diagram (Figure 1). The authors (HP and MB) evaluated the same 685 articles after seven duplicates were removed. Based on inclusion and exclusion criteria, 654 articles were excluded and 50 were sought for retrieval. Forty-eight were obtained and assessed for eligibility. Authors (HP and MB) independently reviewed each full-text publication. Upon further review, 15 articles were excluded due to the wrong population. The wrong population was defined as studies, not about patients with SARS-CoV-2 and gout. One review article was excluded, 11 articles were out of scope, and one was excluded due to an incorrect outcome. Disagreements were resolved by discussion with all authors yielding 20 total studies included in the analysis. Additional reference lists were not utilized. 

Data Collection 

Authors (HP and MB) evaluated each article and developed a data abstraction table including author, year of publication, country of study, study design, sample size, outcomes, and limitations of the study. Once data were collected and a qualitative analysis was performed on the data table, the narrative review was written.

Results****

Impact of COVID-19 on Gout Flares, Diagnosis, and Management 

The COVID-19 pandemic changed the accessibility of healthcare and had significant implications on the diagnosis and treatment of many conditions. Specifically, the pandemic was associated with a decreased incidence of gout diagnosis. In addition, patients with gout experience an increased number of gout flares. Furthermore, patients with gout experienced higher urate levels. Lifestyle changes and increased fear associated with seeing physicians due to fear of contracting COVID-19 infection led to worsening of chronic diseases such as gout [8-11]. In one study, the investigators demonstrated patients with gout had nine times more gout flares and higher levels of urate during the pandemic [10]. This study only explored patients during the pandemic, it did not specify whether or not they had an active COVID-19 infection when discussing gout flares [10]. It can be hypothesized that higher levels of urate leading to increased gout flares during the pandemic were due to decreased use of urate-lowering therapies due to the fear of seeing physicians during the pandemic [9]. In addition, the diagnosis of gout and other rheumatic diseases declined during the pandemic due to lockdowns, social distancing, and patient concerns about coming into physician offices [11].

Factors Impacting Outcomes in Patients With Comorbid Gout and SARS-CoV-2 Infection

Women as compared to their male counterparts experience lower rates of severe infection and mortality tied to SARS-CoV-2 infection, especially when vaccinated [8,9,12-15]. Another important consideration in prognosis alongside uric acid levels is gender. Male patients had lower uric acid/creatine (UA/Cr) levels in urine than females while females had lower serum uric acid levels [4,13,16-19]. The cut-off values for uric acid were found to be 5.15 mg/dL and for creatinine were found to be 0.9 mg/dL [20]. A potential reason for higher serum uric acid in males is due to increased testosterone which upregulates URAT1, increasing reabsorption of uric acid, thus increasing serum uric acid levels [19]. The female immune system creates a greater amount of CD4 T cells than males, thus, males possibly require more ICU care as there is potential for a decreased immune response from a decreased amount of CD4 T cells in comparison to females [20]. Upon discharge, it was noted males had low serum uric acid levels as well as low UA/Cr levels [19]. On admission, low serum uric acid and low UA/Cr were found to lead to worsened SARS-CoV-2 outcomes and subsequently increased mortality [4,19].

In individuals with comorbidities such as gout, vaccination is important as it has been shown to lower the rates of hospital admission and mortality in comparison to unvaccinated individuals [16]. Rheumatic diseases such as gout had an increased risk of hospital admission and mortality compared to many other rheumatic diseases, showing the importance of vaccination in gout patients [16]. Therefore, it is important for physicians to focus on vaccinating individuals suffering from rheumatic diseases and connective tissue diseases as vaccination is shown to decrease SARS-CoV-2 severity and mortality [12,16].

In addition, pharmaceutical drugs used to treat Gout including the use of long-term glucocorticoids, DMARDs, a prednisone dose of 10 mg or more a day, rituximab, sulfasalazine cyclophosphamide, and mycophenolate mofetil are also risk factors for increased hospitalization stay and mortality in patients with SARS-CoV-2 patients and gout [16,17].

Influence of SARS-CoV-2 Infection on Gout

SARS-CoV-2 infection appears to have a greater prevalence in individuals with co-morbidities due to an inadequate immune response causing worsened infection with SARS-CoV-2 infection [13]. It has been demonstrated that patients with rheumatic conditions including gout and other conditions experienced higher rates of mortality tied to SARS-CoV-2 infection [7]. Specifically, gout patients with SARS-CoV-2 had greater needs for ventilatory support and oxygen therapy [7]. Patients with gout had poorer outcomes with SARS-CoV-2 infection due to increased urate levels causing a heightened inflammatory condition in comparison to healthy patients, who have normal levels of urate. Uric acid activates several intracellular signaling pathways, which results in the production of inflammatory cytokines, tissue factors, and chemokines thus precipitating further inflammation [15].

Patients with gout and co-morbid COVID-19 infection have been shown to experience higher rates of 30-day hospitalizations, an increased 30-day mortality rate, and more breakthrough infections as compared to patients without gout as a risk factor. It is possible the innate immune system is impaired in gout patients leading to increased susceptibility to infection and worse outcomes. Xie et al. suggest that increased uric acid levels suppress neutrophil adhesion and extravasation in mice with SARS-CoV-2 inflammation or infection [15]. In addition, IL-6 levels are higher in patients with rheumatic diseases such as gout, increasing the number of recruited lymphocytes to the synovial fluid when infected with SARS-CoV-2, leading to greater inflammation and worsening outcomes [1].

Uric Acid as a Prognostic Factor of SARS-CoV-2 Mortality

**Figure 2:**
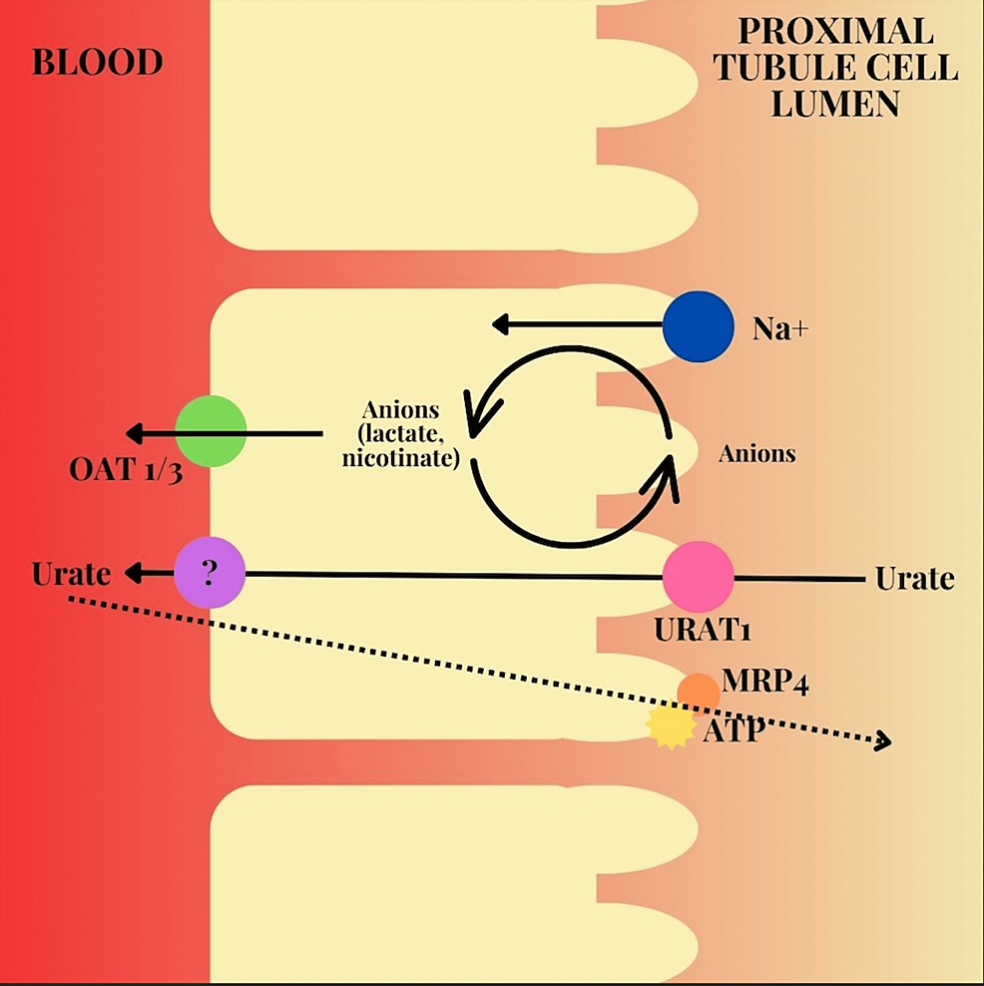
Urate transporter mechanism: Urate transporter 1 gene (URAT1) is a protein that regulates uric acid levels in the body. The figure above outlines how URAT1 facilitates the reabsorption of uric acid from urine back into the bloodstream. Uric acid is a waste product during the breakdown of purines. Elevation of uric acid levels lead to conditions such as gout. Image Credits: Mahi Basra, Author

The level of serum uric acid may serve as a prognostic indicator of poor outcomes and increased mortality risk from SARS-CoV-2 infection among patients with gout. Uric acid is filtered by the proximal tubule of the kidney via the urate transporter (Figure 2). SARS-CoV-2 disrupts this process by downregulating the expression of urate transporter 1 gene (URAT1), leading to lower uric acid levels from a defect in reabsorption of uric acid, causing hypouricemia and uricosuria [17,18]. It has been shown that patients with gout who become infected with severe SARS-CoV-2 infection were found to have lower uric acid levels, while those patients with gout with mild SARS-CoV-2 infection had higher uric acid levels. Although gout is caused by increased levels of serum uric acid, some studies suggest that SARS-CoV-2 lowers uric acid levels. Other studies mention a U-shaped distribution. The change in uric acid is described in a U-shaped manner with the low being less than 278 µmol/L of serum uric acid and the high being greater than 423 µmol/L of serum uric acid [2]. As such, a measure of uric acid levels may serve as a prognostic indicator of the potential severity of SARS-CoV-2 infection among patients with gout. Meanwhile, the underlying mechanisms tied to SARS-CoV-2 infection and gout flares remain unclear and warrant further investigation.

The impaired pulmonary function in SARS-CoV-2 causes hypoxia in tissues. This can lead to a decrease in oxygen uptake and a decrease in uric acid levels, further increasing inflammatory markers such as IL-6 and C-reactive protein (CRP) [20]. IL-6 is an important proinflammatory mediator as it is elevated in gout flares and is noted to be elevated in SARS-CoV-2 infection [21]. This alteration in uric acid levels about SARS-CoV-2 infection shows the potential for uric acid levels to be used as a prognostic indicator as it can identify mild versus severe infection.

Uric acid is known to be an antioxidant that helps to restore the function of endothelial cells and protect the CNS at low to normal levels [19]. In patients with severe hypouricemia (normal range = 3.5 to 7.2 mg/dL, hypouricemia = below 3.4 mg/dL), patients may present with endothelial dysfunction, reduced blood pressure, and reduced defense against reactive oxygen species (myeloperoxidase activity and increased lipid peroxidation), which are associated with poorer SARS-CoV-2 outcomes [17]. Uric acid is known to modulate the innate immune system, so low levels can further exacerbate SARS-CoV-2 infection by modulating a cytokine storm [17]. When uric acid levels are increased, patients appear to be at increased risk for infections such as respiratory syncytial virus, cystic fibrosis, lung disease, and sepsis as well as gout, insulin resistance, cardiovascular disease, and death [19]. In addition, uric acid is known as an antioxidant that helps protect neurons from oxidative stress. When levels of uric acid are too low, it leads to disruption of neurons causing Alzheimer’s disease, Guillain-Barre syndrome, and meningitis [19]. Additional research is needed to fully understand the mechanism by which uric acid levels influence neuronal diseases.

**Figure 3:**
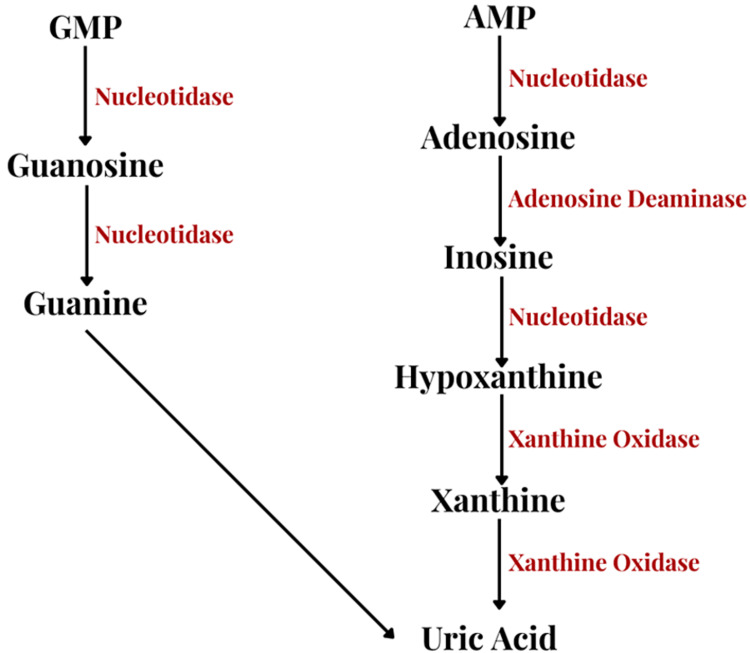
Uric acid metabolism pathway: Uric acid is a waste product in the breakdown of purines (adenosine and guanine). Purines are converted to xanthine which is metabolized to uric acid. Uric acid is then transported via URAT1 through the bloodstream to the kidneys, reabsorbed in the bloodstream or excreted as urine. Image Credits: Mahi Basra, Author

Meanwhile, uric acid is an antioxidant that is used by oxidizing agents to prevent inflammatory reactions, which can then cause higher levels of morbidity of SARS-CoV-2 infection [12,19]. In addition, in the uric acid metabolism pathway, as seen in Figure 3, guanosine monophosphate (GMP) is metabolized into uric acid and excreted from the body [19]. In SARS-CoV-2 patients, GMP levels are low, leading to lower uric acid levels. Furthermore, CD39 and CD73 are inflammatory mediators that require adenosine triphosphate (ATP) to adenosine monophosphate (AMP) dephosphorylation for energy [19]. AMP is a part of the uric acid metabolism pathway as well and low levels are found in SARS-CoV-2 patients.

Other Prognostic Factors of SARS-CoV-2 Mortality

Another predictor of COVID-19 outcome is changes in serum calcium levels. Calcium levels were found to be low (less than 8.8 mg/dL) in mild/moderate groups in the early stages (1-28 days), while late stages (after 28 days) showed normal calcium levels (8.8 to 10.3 mg/dL). In severe and/or critical patients, calcium levels were always lower than in the mild/moderate group in the early stages of infection [22]. In addition, hypocalcemia in patients with COVID-19 infection can be attributable to viral E gene entering host cells [22]. The viral E gene encodes for a transmembrane protein that functions as a calcium ion channel, responsible for calcium homeostasis [22]. This alteration in homeostasis leads to inflammation and lung damage, worsening SARS-CoV-2 severity. Meanwhile, many studies do not indicate sufficient results in the same way that uric acid acts as a prognosis factor does. Thus, calcium levels can be used in conjugation with uric acid as a prognostic factor.

Viral infections can lead to a robust immune response, activating many inflammatory mediators, leading to a large production of reactive oxygen species that induces the secretion of more proinflammatory mediators to remove the virus.

There are many factors contributing to the increased mortality in SARS-CoV-2 patients such as older age causing a decline in immune function, male gender, and high levels of uric acid, creatinine, and potassium [20]. In addition, high levels of high-sensitivity troponin, ferritin, CRP, LDH, D-dimer, and lymphopenia all can predict severity [20]. Furthermore, certain disease states alter the level of uric acid that leads to worsening SARS-CoV-2 whichoutcomes. In patients with acute respiratory distress syndrome (ARDS) which is defined as lung failure secondary to fluid buildup and low oxygen, uric acid levels greater than 8.4 mg/dL had the highest mortality, and high levels of uric acid were also found in patients with chronic obstructive pulmonary disease (COPD), pulmonary hypertension, obstructive sleep apnea, hypertension, cardiovascular disease, atrial fibrillation, non-fatty liver disease, and chronic kidney disease [20]. In patients with gout or treated with thiazide diuretics, low uric acid levels were found [20]. Thus, uric acid shows promise to be used as a prognosis indicator in patients with rheumatic diseases infected with SARS-CoV-2. The results table summarizes all the articles (Table 1).

**Table 1:** Summary of Articles

Reference	Study Design	Data Collection	Study Aim	Findings	Limitations
Association of Serum Calcium and Serum Uric Acid Level with Inflammatory Markers to Predict the Outcome of COVID-19 Infection: A Retrospective Study [22]	Retrospective Study	N = 136 patients Serum calcium and uric acid levels were collected from patients Descriptive statistics, ANOVA and t-Test were utilized	Assess correlation of serum calcium and uric acid levels with inflammatory markers and outcome of SARS-CoV-2	Early serum Ca2+ and uric acid levels can help predict disease outcome Hypocalcemia in SARS-CoV-2 patients can be due to viral E gene entry in host cells which may lead to inflammation and lung damage Due to viral inflammation, uric acid levels are increased which can act as a reactive oxygen species (ROS)	Small sample size Corrected serum calcium and uric acid levels were not considered in patients
Association of serum uric acid levels with COVID-19 severity [19]	Case Control Study	N = 91 patients with 273 age/sex matched controls Serum uric acid (SUA) and uric/acid creatinine ratio were compared between SARS-CoV-2 and healthy patients	Investigate whether SARS-CoV-2 patients had decreased SUA levels and assess relationship with severity of SARS-CoV-2	Serum Uric Acid (SUA) levels in SARS-CoV-2 patients were 2.59% lower when compared to healthy controls UA/Cr ratios were 6.06% lower when compared to healthy controls SUA levels and UA/Cr were lower in male patients Male patients who experienced increased severity of symptoms had lower SUA	Small sample size
Characteristics and Outcomes of People With Gout Hospitalized Due to COVID-19: Data From the COVID-19 Global Rheumatology Alliance Physician-Reported Registry [7]	Retrospective Study	N = 163 patients with gout who developed SARS-CoV-2 and were hospitalized	Characterize outcomes of patients hospitalized with SARS-CoV-2 in 2019	46% of the patients hospitalized had 2+ comorbidities 68% of patients required supplemental oxygen or ventilation during hospital stay	Small sample size
Clinical outcomes of COVID-19 patients with rheumatic diseases: a retrospective cohort study and synthesis analysis in Wuhan, China [1]	Retrospective Cohort Study	N = 4353 SARS-CoV-2 patients Statistical analysis was done on patients after collecting data	Characterize clinical characteristics of SARS-CoV-2 patients	Rheumatic diseases (RD) aggravate SARS-CoV-2 infection Poor outcomes of SARS-CoV-2 are not associated with RD Higher levels of IL-6 noted in RD patients	Sample size of rheumatic disease group was smaller than control group Lab tests were not performed for all patients
Coronavirus disease 2019 (COVID-19) infection in patients with rheumatic diseases: Clinical characteristics and relation to anti-rheumatic therapy [12]	Retrospective study	N = 215 RD patients Statistical analysis was done on patients after collecting data	Describe manifestations of SARS-CoV-2 in patients with rheumatic disease and investigate relationship with antirheumatic therapy	Body aches was most common SARS-CoV-2 symptom mainly in RD patients Patients who had infection were older and had other comorbidities No difference noted in treatment type of RD patients Prednisone use worsened in COVID and RD if they had prolonged intake of steroids Anti-TNF-alpha treatment increased SARS-CoV-2 infection risk No change noted between use of steroids, immunosuppressants, glucocorticoids, and csDMARD	Lack of healthy control group Small sample size from single center
Death due to COVID-19 in a patient with diabetes, epilepsy, and gout comorbidities [13]	Case Report	N = 1 patient	Describe case of patient with acute respiratory distress syndrome	SARS-CoV-2 and comorbidities like DM and epilepsy and gout lead to multiple organ failure, death, ICU admission	N/A
Gout and Excess Risk of Severe SARS–CoV-2 Infection Among Vaccinated Individuals: A General Population Study [15]	Retrospective Cohort Study	Health Improvement Network was utilized (UK) and comparison of risk of breakthrough infection, hospitalization and death between individuals with gout	Examine association between gout and SARS–CoV-2 infection	Women had higher incidence of SARS–CoV-2 infection and had more severe outcomes Gout patients with comorbidities lead to higher SARS–CoV-2 infection risk 30 day hospitalization rate and death was higher in patients with gout and SARS–CoV-2	Gout patients are more likely to seek medical care than control group
Gout and the risk of COVID-19 diagnosis and death in the UK Biobank: a population-based study [14]	Case Control Study	N = 15,871 from UK Biobank Multivariable logistic regression was used to test for association	Assess whether gout is a risk factor for SARS–CoV-2 diagnosis and death	Gout is a risk factor for SARS–CoV-2 death especially in women and patients with comorbidities No differences noted in risk of SARS–CoV-2 related deaths in relationship to urate therapy or colchicine	Data only derived from middle aged White British ethnic group Potential unidentified SARS–CoV-2 related deaths SARS–CoV-2 outcomes may have been affected over the period of the study Flawed reporting in biobank Non-SARS–CoV-2 related risk of death was not accounted for Severity of gout could not be assessed Compliance of patients with medication was not assessed nor was individual behavior
Gout during the SARS-CoV-2 pandemic: increased flares, urate levels and functional improvement [10]	Cohort study	Primary care and hospital EHR data for 17.9 million adults was used via OpenSAFELY	Asses how SARS–CoV-2 pandemic impacted incidence and quality of care for patients with gout in England	Gout flares and higher urate levels were seen during the pandemic Flares were uncontrolled when infected with SARS–CoV-2	Only 80% of pre-pandemic patients could be located with scheduled visits Lifestyle changes were not evaluated
Gout incidence and management during the COVID-19 pandemic in England, UK: a nationwide observational study using OpenSAFELY [9]	Cohort study	Primary care and hospital EHR data for 17.9 million adults was used via OpenSAFELY	Asses how SARS–CoV-2 pandemic impacted incidence and quality of care for patients with gout in England	There were reduced gout diagnoses during the pandemic The pandemic affected patients with long term health issues There was no increase in gout diagnosis Several patients remained undiagnosed during the pandemic	Potential diagnosis misclassification when using coded health data
Gout, Rheumatoid Arthritis, and the Risk of Death Related to Coronavirus Disease 2019: An Analysis of the UK Biobank [5]	Case Control Study	UK Biobank data: hospital diagnosis 1991-June 30, 2020, SARS-CoV-2 test between March 16-August 24, 2020 and death records up until August 14, 2020 Multivariable- adjusted logistic regression: analysis A- gout and/or RA and COVID-19 diagnosis (n=473,139), analysis B- gout and/or RA and death from COVID-19 (n= 2059), analysis C- gout and/or RA and death from COVID-19 in entire UK Biobank cohort (n= 473,139)	Assess whether gout and/or RA are risk factors for COVID-19 diagnosis and COVID-19 death	RA is a risk factor for death from COVID-19	Need larger data set that allows inclusion of wider range of factors
Hyperuricemia and Adverse Outcomes in Patients Hospitalized for COVID-19 Disease [4]	Retrospective Study	Patients hospitalized for COVID-19 between March 15-November 30, 2020 (n= 1566) at Shaare Zedek Medical Center that had uric acid blood test first 4 days of admission Norton scale score (NSS) on admission to evaluate condition of patient: 4-20 range, assess physical and mental conditions, activity, mobility and incontinence	Determine if hyperuricemia is a risk factor for COVID-19 related death or other adverse outcomes	High uric acid associated with adverse outcomes from COVID-19	Small sample size, unavailable uric acid levels in 80% of patients with COVID-19, blood samples not drawn at the same time on admission in each patient
Incidence of rheumatic diseases during the COVID-19 pandemic in South Korea [11]	Retrospective Study	January 2016-December 2020 patients from Korean Health Insurance Review and Assessment Service database Autoregressive integrated moving average (ARIMA) and quasi- Poisson analyze used to analyze disease incidence before and after the COVID-19 pandemic	Incidence of RA, SLE, gout, BD and AS during the COVID-19 pandemic in South Korea	IIM, SLE, BD, AS, and gout decreases within the first half of 2020	Disease severity may not be accurately reflected from the registered diagnosis code, 1 year is too short to evaluate changes in incidence of chronic diseases, National Health Insurance states database lacked accurate clinical information on individual patients
Omicron variant infection in inflammatory rheumatological conditions – outcomes from a COVID-19 naive population in Aotearoa New Zealand [16]	Observational Study	People with inflammatory rheumatic disease and SARS-CoV-2 infection in Aotearoa New Zealand between February 1-April 30, 2022 Data collected via the Global Rheumatology Alliance Registry which included demographics, disease characteristics and COVID-19 vaccination and outcome	Outcome of SARS-CoV-2 Omicron variant in inflammatory rheumatic disease patients	Inflammatory rheumatic diseases and high vaccination rates led to less frequency of severe outcomes from SARS-CoV-2 Omicron variant	Lack of control group, not generalized to the general public to compare to, cannot represent general population, patients who died outside of hospital did not report cause of death because primary care records not included
Outcomes of COVID-19 in people with rheumatic and musculoskeletal disease in Ireland over the first 2 years of the pandemic [23]	Retrospective Study	Data from the C19-GRA provider registry from Ireland between 24th March 2020 and 31st March 2022 (n= 237)	Analyze the COVID-19 outcomes of the first two years of the pandemic for people with rheumatic musculoskeletal diseases (RMD) in Ireland over	Hospitalization or death more frequent in RMD patients with comorbidities, older age, certain medication which are indicators to help the management of RMD patients	Low case number, selection bias
Serum Uric Acid Concentrations and Risk of Adverse Outcomes in Patients With COVID-19 [21]	Retrospective Study	Patients with confirmed COVID-19 admitted to Leishenshan Hospital(n=1854) Logistic regression analysis to explore association between uric acid concentration and COVID-19 adverse outcomes	Relationship of serum uric acid on admission in hospitalization patients with COVID-19	Admission serum uric acid and COVID-19 outcome is U-shaped: baseline uric acid levels are 279-422 µmol/L, and values greater than or equal to 423 µmol/L or less than or equal to 278 µmol/L associated with more adverse outcomes	No causality established between serum, uric acid and mortalities, ICU admission and ventilation required and no uric acid follow up measurements
Serum uric acid, disease severity and outcomes in COVID-19 [17]	Retrospective Study	Patients admitted to the Cliniques universitaires Saint- Luc, Brussels, Belgium, with SARS-CoV-2 during the February 23, 2020 to April 18, 2020 and September 21, 2020 to November 21, 2020 waves of the pandemic. Cox regression used to analyze survival and confocal microscopy to assess URAT1 in the kidney of those who died from COVID-19	Low serum uric acid may lead to more severe COVID-19 outcomes	Low serum uric acid is associated with severe disease and progression to need for mechanical ventilation	Single design study, lack of follow up and lack of insight on the pathophysiology linking hypouricemia and respiratory failure
Uric Acid and Mortality Relationship in COVID-19 [20]	Retrospective Study	397 patients with COVID-19: 157 deceased, 225 ICU admission, 240 recovered Patients from the internal medicine clinic with symptomatic pneumonia between April 15, 2020-December 15, 2020	Compare uric acid of recovered and decreased COVID-19 patients and the ICU of COVID-19 patients	The cutoff value of uric acid that may pose a risk of mortality was 5.15 mg/dL and was 0.9 mg/dL for creatinine. Advanced age, increased creatinine and increased potassium associated with death	None stated
Uric acid as a prognostic predictor in COVID-19 [18]	Retrospective Study	COVID-19 admitted patients from March 2020-March 2021, uric acid levels on admission for 498 patients and severe period of COVID-19 in 143 patients Chi-square method, T-test and ANOVA test for differences in variables, correlation tests	Investigate incidence and prognostic significant of baseline and control uric acid values in COVID-19 patients	Low uric acid levels had increased ICU admission and decline in uric acid during stay at the hospital predicted a poor prognosis	Lack of data of uric acid levels of patients prior to infection, no measure of urinary uric acid
U-shaped association between abnormal serum uric acid levels and COVID-19 severity: reports from the Japan COVID-19 Task Force [24]	Retrospective Study	1523 patients in the Japan COVID-19 Task Force between February 2020-May 2021 Lab results collected within 48 hours of initial visit or admission	Relationship between abnormal serum uric acid levels or history of hyperuricemia and COVID-19 disease severity	Abnormal serum uric acid levels, high or low, demonstrating a U-shaped curve as well as a history of hyperuricemia is associated with COVID-19 severity	Lower prevalence of hyperuricemia than the actual prevalence in the population, same for history of CKD, no record of current medications, no following of serum uric acid levels during hospitalization

Discussion

Gout occurs due to an inflammatory reaction leading to accumulation of uric acid crystals [2,3]. Gout patients experienced higher levels of urate due to changes in lifestyle and increased fear of seeing physicians during the pandemic. This may have been due to a decreased use of urate-lowering therapies due to fear of the infection. Additionally, because access to healthcare was altered during the pandemic, there were significant implications on diagnosis and treatment of many conditions. Specifically, the pandemic was associated with a decreased incidence of gout diagnosis. In addition, patients with gout experience an increased number of gout flares. Furthermore, patients with gout experienced higher urate levels. Changes in lifestyle and increased fear associated with seeing physicians due to fear of contracting SARS-CoV-2, led to worsening of chronic diseases such as gout. Gout is linked to several comorbidities including obesity, T2DM, hypertension, hyperlipidemia, chronic kidney disease, chronic heart disease, cardiovascular disease, and heart failure, all of which may further worsen COVID-19 infection [13]. In addition, the infection is also more common among older patients of female gender.These patients with multiple comorbidities experienced greater need for ventilatory support, rates of hospitalization, mortality rate and breakthrough infections [7].

There are many comorbidities associated with gout, such as increased rates of mortality when infected with SARS-CoV-2. It was concluded that women with gout had lower rates of severe SARS-CoV-2 infection due to an increased immune response from higher levels of CD4 T cells in comparison to men [4,14,15,19]. It can be concluded that the higher levels of testosterone in males upregulates URAT1, increasing reabsorption of uric acid and therefore increasing uric acid levels [19].

Due to the change in uric acid in patients with gout and COVID-19 infection, uric acid levels have the potential to be used as a predictor marker of COVID-19 infection severity in gout patients. Patients with gout who experienced more severe infection were found to have lower levels of uric acid and those patients with higher uric acid levels experienced milder infection. This distribution is known as a U-shaped curve: mild infection had higher uric acid levels and severe infection had lower uric acid level [2,17,18]. The cut off values were greater than 423 µmol/L of serum uric acid and less than 278 µmol/L of serum uric acid [2,17,18]. When uric acid levels fell below or above 278-423 µmol/L, there was an increased risk of poor outcomes in SARS-CoV-2 patients [2,17,18]. An additional prognostic factor that can help determine outcome of COVID-19 infection is serum calcium levels. In mild/moderate COVID-19 infection, serum calcium levels were low. In severe/critical COVID-19 infection, calcium levels were significantly lower than the mild/moderate COVID-19 infection group [22]. Future studies should focus on the exact cut-off values determining mild, moderate, severe and critically ill COVID-19 infection.

Strengths and limitations

The Preferred Reporting Items for Systematic Reviews and Meta-Analyses (PRISMA) flow diagram shown in Figure 1 portrays the process of data collection and inclusion of articles for this review. A total of 20 articles were chosen from 685 total publications. Strengths of this study include the use of reproducible methodology and a comprehensive search performed of relevant articles. This provided data with high specificity and reproducibility. Only relevant literature from the last 10 years was included to keep data current and up to date. 

A potential limitation of this study is that some studies on the relationship between gout and COVID-19 infection may have been missed due to our inclusion and exclusion criteria such as only including studies in the English language that have been peer reviewed and those published from 2013 onward, despite our team conducting an extensive search using multiple databases.

It is important to note some of the limitations of included articles. Small sample sizes were noted in six studies, as can be seen in Table 1, which may restrict the generalizability of the findings [4,7,12,19,22,23]. One study by Feldman et al. was unable to obtain uric acid levels in 80% of patients with COVID-19 infection. The authors noted that samples were not drawn at the same time on admission in each patient, which may have led to some confounding factors [4]. Another study performed by Kruthi et al. did not consider corrected serum calcium and uric acid levels in patients [22], and the study conducted by Chen et al. did not take into account causality between serum uric acid levels and mortalities [21]. In addition, there was a lack of uric acid measurements in follow-up [18,21,24]. A study by Fukushima et al. did not record current medications in patients, and their study population had a lower prevalence of hyperuricemia and chronic kidney disease than the general population, which makes the results difficult to generalize [24]. Dufour et al. aimed to conduct a study correlating low serum levels of uric acid to severity of COVID-19. However, a clear pathophysiology link between hyperuricemia and respiratory failure was not made. There was also a lack of patient follow-up [25].

In the study performed by Qi et al., the control group sample size was smaller than the original sample size of patients with rheumatic disease. All lab tests were also not performed on each patient [1]. In the study performed by Aboud et al., there was no healthy control group. Furthermore, the study had a relatively small sample size. As a result of this study being a retrospective observational study, it is hard to accurately draw conclusions and comparisons to the manifestations of COVID-19 infection in patients with no rheumatic disease [2]. Comparably, the observational study by Brooks et al. lacked a control group, thus making it difficult to generalize the results to the population [16]. Garcia-Maturano et al. were unable to assess the impact of lifestyle changes, which may help to inform future studies on other factors affecting prognosis and morbidity [10].

Although Xie et al. utilized the Health Improvement Network in the United Kingdom (UK), allowing for a database representative of the population, one limitation was that the study authors noted that gout patients included in the study may have sought medical care more often than controls during the pandemic thus overestimating the risk of COVID-19 infection [15]. Similarly, although Topless et al. utilized the UK Biobank, the data were only derived from the middle age white British population, thus leaving potential unidentified SARS-CoV-2 infection-related deaths. Non-SARS-CoV-2 infection-related deaths were not accounted for either and severity of gout was not assessed. OpenSAFELY health record software was used in the study by Russel et al., which allowed for assessment of 17.9 million adults in the UK. Meanwhile, even with this database, there is a potential that diagnoses were misclassified using coded health data leading to some unreported cases [9]. Likewise, in the study by Ahn et al. evaluating disease severity in the Korean Health Insurance Review and Assessment Service Database may have not been accurately reflected due to registered diagnosis codes. In addition, it is difficult to evaluate changes in incidence in chronic diseases over the course of one year [11]. There are limited studies available to correlate gout, uric acid levels and COVID-19 infection. 

## Conclusions

Overall, it can be seen that the pandemic had a clear effect on gout patients. Patients may have experienced worsened outcomes due to COVID-19 infection or may have been fearful to seek treatment due to the risk of exposure. Levels of uric acid were correlated to the severity of COVID-19 infection. Due to the limited amount of studies available that discuss the incidence of gout, uric acid levels, and severity of COVID-19 infection, it is difficult to draw conclusions. However, through the limited literature available, it can be seen that the COVID-19 pandemic significantly altered access to healthcare. Thus, further worsening patient outcomes in those with preexisting or new diseases, such as gout. Uric acid has the potential to be used as a prognostic indicator in those infected with SARS-CoV-2, though several future studies may have to be done to commonly utilize this. 

Future implications

Further studies should be performed on patients with other rheumatic diseases such as ankylosing spondylitis, rheumatoid arthritis, osteoarthritis, and systemic lupus erythematosus to determine if a link exists between SARS-CoV-2 infection and disease course. Additionally, utilizing uric acid as a prognostic factor in patients who do not have gout may assist in shedding some light on the significance of its levels. Studies can also be stratified by region to assess other environmental factors that may affect patients. Additional primary studies should also consider the patients’ lifestyle factors. Metabolites in the purine synthesis pathway such as xanthine and hypoxanthine levels may also aid in further stratifying prognostic factors. There are no animal or in vitro studies to explain the mechanism of action of rheumatic diseases and SARS-CoV-2 mortality, which can be a focus of future studies. Overall, there is a clear link between gout, uric acid levels, and SARS-CoV-2 as highlighted by this scoping review. 

Rheumatic diseases can lead to chronic inflammation from various regions of the body. A common rheumatic disease is gout, which is an inflammatory joint disease that can lead to joint destruction when untreated. There are many associated comorbidities such as T2DM, epilepsy, hypertension, hyperlipidemia, chronic kidney disease, and cardiovascular disease. In gout, there is a high level of serum uric acid. When a gout patient is infected with SARS-CoV-2, there is a heightened immune response and change in uric acid level. Additionally, studies indicate a U-shaped pattern in which significantly decreased levels of uric acid are linked to severe SARS-CoV-2 infection. In contrast, significantly increased levels of uric acid are associated with mild SARS-CoV-2 infection. This scoping review analyzed the relationship between SARS-CoV-2 infection and uric acid levels in individuals with gout. There were many limitations to the study that included limited article inclusion, small sample size, and lack of properly documented SARS-CoV-2 vs non-SARS-CoV-2 related deaths. Lastly, individual diet and exercise should be accounted for. A diet of alcohol and red meat can worsen gout with SARS-CoV-2 or not, which is an important variable to control.
